# Classification of glucose profile in Japanese patients with type 1 diabetes and its association with complications

**DOI:** 10.1210/clinem/dgag224

**Published:** 2026-06-08

**Authors:** Takafumi Masuda, Naoto Katakami, Naohiro Taya, Kazuyuki Miyashita, Mitsuyoshi Takahara, Ken Kato, Iichiro Shimomura

**Affiliations:** Department of Metabolic Medicine, Osaka University Graduate School of Medicine, Suita City, Osaka 565-0871, Japan; Department of Metabolic Medicine, Osaka University Graduate School of Medicine, Suita City, Osaka 565-0871, Japan; Department of Metabolic Medicine, Osaka University Graduate School of Medicine, Suita City, Osaka 565-0871, Japan; Department of Diabetes Care Medicine, Osaka University Graduate School of Medicine, Suita City, Osaka 565-0871, Japan; Department of Metabolic Medicine, Osaka University Graduate School of Medicine, Suita City, Osaka 565-0871, Japan; Department of Metabolic Medicine, Osaka University Graduate School of Medicine, Suita City, Osaka 565-0871, Japan; Department of Laboratory Medicine, Osaka University Graduate School of Medicine, Suita City, Osaka 565-0871, Japan; Diabetes Center, NHO Osaka National Hospital, Chuo-ku, Osaka City, Osaka 540-0006, Japan; Department of Metabolic Medicine, Osaka University Graduate School of Medicine, Suita City, Osaka 565-0871, Japan

**Keywords:** atherosclerosis, cluster analysis, continuous glucose monitoring, microangiopathy, type 1 diabetes

## Abstract

**Context:**

The importance of utilizing continuous glucose monitoring (CGM) to optimize glycemic profiles and thereby prevent the onset of diabetic complications in patients with type 1 diabetes has been increasingly recognized. Nevertheless, studies evaluating the risk of diabetic complications in patients with type 1 diabetes through machine learning approaches based on CGM data remain limited.

**Objective:**

To classify the glycemic profiles of Japanese patients with type 1 diabetes using a data-driven cluster analysis based on CGM and clarify the association between these clusters and diabetic complications.

**Methods:**

In this cross-sectional study, a cluster analysis using glycemic metrics from CGM of 153 Japanese patients with type 1 diabetes was performed. Logistic regression analysis adjusted for age, sex, and duration of diabetes was performed to compare the risk of diabetic complications by cluster.

**Results:**

The cluster analysis identified four clusters. Cluster 1 (n = 53) exhibited an optimal glycemic profile. Cluster 2 (n = 46) demonstrated an extended duration of hyperglycemia and a higher risk of elevated brachial–ankle pulse wave velocity than Cluster 1. Cluster 3 (n = 39) demonstrated an extended duration of hypoglycemia and a higher risk of severe hypoglycemia than Cluster 1. Cluster 4 (n = 15) demonstrated large glycemic variability associated with hyperglycemia and hypoglycemia. Cluster 4 had higher risks of polyneuropathy, elevated brachial–ankle pulse wave velocity, and higher cardiovascular disease risk scores than Cluster 1.

**Conclusion:**

High-risk diabetic complications were identified for each cluster classified by glycemic profile.

Improvement of glycemic profile is increasingly being emphasized in diabetes management. In addition to controlling glycated hemoglobin (HbA1c), suppressing glycemic variability is crucial for preventing diabetic complications (1). Continuous glucose monitoring (CGM) has become widely utilized in the management of diabetes, particularly type 1 diabetes, whose onset is primarily driven by pancreatic β-cell dysfunction. In patients with type 1 diabetes, the lack of endogenous insulin makes glycemic control challenging, and evaluation of glycemic profiles based on CGM provides substantial support for glycemic control.

Management targets for individual glycemic variability metrics derived from CGM have been established in international consensus statements (2), and achieving these goals is crucial for preventing the onset of diabetic complications. Glycemic variability metrics obtained from CGM are associated with the risk of diabetic complications in type 1 diabetes (3-8), highlighting the importance of treatment approaches that consider the effects of glycemic variability on diabetic complications. Accordingly, optimal therapeutic strategies should be established for patients with type 1 diabetes based on CGM-derived glycemic profiles.

The use of machine learning to develop individualized treatment strategies for patients with diabetes is gaining attention. However, reports on the application of machine learning approaches to evaluate the risk of diabetic complications based on glycemic profiles derived from CGM data remain limited. In a previous study in India that employed non-negative matrix factorization to classify patients with type 2 diabetes into three glycemic profiles using eight CGM-derived metrics of glycemic variability, the risk of diabetic complications differed among the profiles (9). Furthermore, a recent study among patients with type 1 diabetes in France demonstrated that by applying discriminative dimensionality reduction with trees using variables such as HbA1c and CGM data, one dimension of a two-dimensional phenotypic tree was associated with retinopathy (10). However, these approaches have primarily been applied to specific ethnic populations and remain to be validated in Japanese patients with diabetes.

This study aimed to classify the glycemic profiles of Japanese patients with type 1 diabetes using a CGM data-driven cluster analysis and to clarify the association between these clusters and diabetic complications.

## Materials and methods

### Study population

This cross-sectional study enrolled a total of 153 patients with type 1 diabetes, who were using CGM and visiting Osaka University Hospital or National Hospital Organization Osaka National Hospital. Eligibility criteria were patients with type 1 diabetes aged 20-80 years at the time of informed consent, and the study enrollment period was from January 2022 to December 2025. Type 1 diabetes was diagnosed by the attending physicians according to the diagnostic criteria of the Japan Diabetes Society (11-13). However, patients with a duration of diabetes of less than 1 year, severe infection or trauma, in the perioperative period, a history of pancreas transplantation, or receiving oral or intravenous steroid therapy were excluded. All procedures were conducted in accordance with the ethical guidelines for medical and biological research involving human subjects in Japan and with the 1964 Helsinki Declaration and its later amendments. The study protocol was approved by the Ethical Review Board of Osaka University Hospital, Japan (approval number: 21378 [T1]), and informed consent was obtained from all participants. The study followed the Strengthening the Reporting of Observational Studies in Epidemiology guidelines (14).

### Patient data and biochemical measurements

The following patient data were collected from electronic health records and questionnaires: age, sex, age at diagnosis, duration of diabetes, smoking status, type of CGM device, use of continuous subcutaneous insulin infusion (CSII), body mass index (BMI), blood pressure, use of statins, use of antihypertensive agents (angiotensin-converting enzyme inhibitors, angiotensin receptor blockers, or calcium channel blockers), use of sodium–glucose cotransporter 2 (SGLT2) inhibitors, history of cardiovascular disease (CVD; acute coronary syndrome, myocardial infarction, stroke, or peripheral artery disease), history of severe hypoglycemia, presence of hypoglycemia unawareness, and treatment satisfaction scores. Severe hypoglycemia was defined as hypoglycemia requiring resuscitative actions of another person. Treatment satisfaction was assessed using the Japanese version of the Diabetes Treatment Satisfaction Questionnaire (DTSQ) (15). The DTSQ comprises eight items, and total satisfaction was calculated as the total score of six questions related to treatment satisfaction (Questions 1, 4, 5, 6, 7, and 8). The remaining two questions (Questions 2 and 3), concerning the perceived frequency of hyperglycemia and hypoglycemia, were evaluated as independent scores.

Biochemical data included serum HbA1c, serum low-density lipoprotein cholesterol (LDL-C), serum triglyceride (TG), urine albumin-to-creatinine ratio (UACR), and estimated glomerular filtration rate (eGFR). All biochemical parameters were measured using standard laboratory protocols, and analyses were performed by SRL Inc. (Tokyo, Japan). LDL-C levels were determined by direct measurement.

### Assessment of glycemic variability metrics

CGM data were collected from Ambulatory Glucose Profile reports using one of the following software platforms according to the CGM device used by each patient: LibreView, Dexcom Clarity, or CareLink. Data were obtained over a 28-day period from LibreView and over a 30-day period from Dexcom Clarity and CareLink. Metrics of glycemic variability included the glucose management indicator (GMI), mean glucose (mean of glucose values), glucose SD, glucose coefficient of variation (CV), time in range (TIR_70–180 mg/dL_), time above range (TAR_>180 mg/dL_, TAR_>250 mg/dL_), and time below range (TBR_<70 mg/dL_, TBR_<54 mg/dL_). The CGM devices were the first-generation or second-generation FreeStyle Libre sensor (Abbott Diabetes Care, Alameda, CA, USA), Dexcom G6 or G7 CGM system (Dexcom, Inc., San Diego, CA, USA), and Medtronic Enlite sensor or Medtronic Guardian sensor 3 or 4 (Medtronic, Northridge, CA, USA). The CGM devices were classified into intermittently scanned CGM (isCGM; first-generation FreeStyle Libre sensor) and real-time CGM (rtCGM; second-generation FreeStyle Libre sensor, Dexcom CGM system, and Medtronic Enlite or Guardian sensor).

### Assessment of microangiopathy

Diabetic retinopathy was evaluated by an ophthalmologist and defined as the presence of at least simple diabetic retinopathy according to the Davis classification (16).

For the assessment of diabetic nephropathy, UACR and eGFR were measured. The eGFR was calculated using the equation for Japanese individuals: eGFR = 194 × serum creatinine^−1.094^ × age^−0.287^ × 0.739 (if female) (17). UACR was calculated from urine albumin and creatinine values obtained from a spot urine sample. Diabetic kidney disease (DKD) was defined as an eGFR < 60 mL/min/1.73 m^2^ or UACR ≥ 30 mg/g.

Polyneuropathy was diagnosed using the simplified diagnostic criteria for diabetic polyneuropathy proposed by the Japanese Study Group on Diabetic Neuropathy (18). In brief, the criteria require the presence of diabetes and the exclusion of other causes of peripheral neuropathy, along with at least two of the following three findings: (i) sensory symptoms suggestive of diabetic polyneuropathy, (ii) decreased or absent bilateral Achilles tendon reflexes, and (iii) decreased vibration sensation in the bilateral medial malleoli.

### Assessment of subclinical atherosclerosis and CVD risk

To assess subclinical atherosclerosis, carotid intima–media thickness (CIMT) and brachial–ankle pulse wave velocity (baPWV) were evaluated. These parameters are established indicators of atherosclerosis and correlate with cardiovascular events and mortality risk (19-21).

CIMT was evaluated using B-mode imaging ultrasonography (Aplio i800 or Aplio i700, TOSHIBA Medical Systems Inc., Tokyo, Japan) with a high-frequency (>7.5 MHz) linear probe based on the Japan Society of Ultrasonics guidelines (22). CIMT was defined as the maximum intimal thickening, including plaques, in the common carotid artery, carotid bulb, or internal carotid artery on longitudinal or transverse views. Measurements were taken bilaterally, and the greater value was used for analysis. The presence of carotid plaque was defined as CIMT ≥ 1.1 mm. All CIMT measurements were performed by a single trained physician.

baPWV was measured using the PWV/ABI device (Omron Colin, Tokyo, Japan), as previously described (20, 23, 24). After at least 5 minutes of rest in the supine position, blood pressure and pulse volume waveforms in both arms and ankles were recorded. baPWV was calculated based on the time difference between the pulse waveforms and the estimated distance between two arterial sites derived from height, with the higher value between the left and right sides adopted for analysis. The presence of elevated baPWV was defined as baPWV ≥ 1400 cm/s, which is reported as the cutoff value for atherosclerotic cardiovascular risk in healthy Japanese subjects (25).

To evaluate the risk of CVD, we used the Steno Type 1 Risk Engine, a risk score model developed to predict the first CVD event in patients with type 1 diabetes (six patients with a prior history of CVD were excluded from the risk score calculation) (26). In this risk score model, a 10-year CVD risk of ≥20% is defined as a high risk for CVD.

### Statistical analysis

Continuous variables are expressed as mean ± SD or median [interquartile range] and categorical variables as frequencies (%). The normality of distribution was visually assessed using histograms and quantile–quantile plots. Variables with a markedly skewed distribution, such as TG and UACR, were log-transformed prior to analysis.

To classify all patients into clusters according to their glycemic profiles, a cluster analysis was performed using the following eight CGM-derived metrics: mean glucose, glucose SD, glucose CV, TIR_70–180 mg/dL_, TAR_>180 mg/dL_, TAR_>250 mg/dL_, TBR_<70 mg/dL_, and TBR_<54 mg/dL_. Although mean glucose and GMI are strongly correlated variables, mean glucose was adopted in the present cluster analysis because it provided superior clustering performance. To classify the study population, we compared the k-means method (analyzed using R statistical software version 4.2.1; R Development Core Team) with the Two-Step clustering method, which combines hierarchical and non-hierarchical clustering approaches. Because the Two-Step clustering method demonstrated superior clustering performance, it was adopted for the present analysis. In the Two-Step clustering method, the optimal number of clusters was first estimated based on silhouette width, followed by hierarchical clustering (27). All eight variables were standardized to a mean of 0 and an SD of 1 before analysis. The optimal number of clusters was automatically determined within a range of 1-15. Two-step clustering was conducted using log-likelihood as the distance measure and Schwarz's Bayesian Information Criterion as the clustering criterion. To ensure appropriate clustering, the average silhouette width was confirmed to be ≥0.5. The cluster with the largest sample size was designated as the reference group for subsequent comparisons. To evaluate cluster stability, we conducted the three analyses presented in the appendix (28).

Unpaired t-tests or chi-square tests were used to compare patient characteristics and the prevalence of microangiopathy (retinopathy, DKD, and polyneuropathy) between clusters. To compare the risk of microangiopathy across clusters, logistic regression analyses were performed for the calculation of adjusted odds ratio (aOR) and 95% confidence interval (CI). Potential conventional risk factors, including age, sex, and duration of diabetes, were included as covariates. To further evaluate the relationships between glycemic variability metrics and markers of subclinical atherosclerosis (CIMT and baPWV), multiple regression analyses were additionally performed using similar covariate adjustments.

All statistical analyses were conducted using IBM SPSS Statistics version 25 (IBM Corp., Armonk, NY, USA). Two-sided *P* values < .05 were considered statistically significant.

## Results

Patient characteristics are presented in Table 1. The patients had a mean age of 48.6 ± 13.5 years, mean diabetes duration of 15.7 ± 12.0 years, mean BMI of 23.4 ± 4.0 kg/m^2^, mean HbA1c of 7.2 ± 0.8%, and mean TIR_70-180 mg/dL_ of 66.4 ± 14.7%. Of the 153 patients enrolled in the study, 66 (43.1%) were male, 107 (69.9%) were using rtCGM, and 92 (60.1%) were using CSII. Diabetic complications included retinopathy in 36 patients (23.5%), DKD in 32 patients (20.9%), polyneuropathy in 39 patients (25.5%), carotid plaque in 70 patients (45.8%), elevated baPWV in 71 patients (46.4%), CVD in 6 patients (3.9%), and severe hypoglycemia in 24 patients (15.7%). Tables S1 and S2 (28) present comparisons of glycemic metrics according to CGM device type and differences in data collection duration.

**Table 1:** Characteristics of all patients (n = 153)

Parameters	
Age (years)	48.6 ± 13.5
Male (%)	66 (43.1%)
Duration of diabetes (years)	15.7 ± 12.0
Type 1 diabetes classification (acute-onset/slowly progressive/fulminant)	102/35/16
Current smoking (%)	28 (18.3%)
Use of rtCGM (%)	107 (69.9%)
Use of CSII (%)	92 (60.1%)
BMI (kg/m^2^)	23.4 ± 4.0
LDL-C (mg/dL)	109.8 ± 27.0
TG (mg/dL)	71.0 (55.0-106.5)
Statin (%)	47 (30.7%)
Systolic blood pressure (mmHg)	128.3 ± 18.0
Antihypertensives (%)	28 (18.3%)
SGLT2 inhibitors (%)	16 (10.5%)
C-peptide (ng/mL)	0.10 ± 0.23
HbA1c (%)	7.2 ± 0.8
GMI (%)	7.1 ± 0.7
Mean glucose (mg/dL)	156.0 ± 25.8
Glucose SD (mg/dL)	56.5 ± 12.9
Glucose CV (%)	36.2 ± 5.9
TIR_70-180 mg/dL_ (%)	66.4 ± 14.7
TAR_>180 mg/dL_ (%)	29.8 ± 15.2
TAR_>250 mg/dL_ (%)	8.4 ± 9.0
TBR_<70 mg/dL_ (%)	3.7 ± 3.6
TBR_<54 mg/dL_ (%)	0.7 ± 1.1
Retinopathy (%)	36 (23.5%)
UACR (mg/g)	5.7 (3.8-11.0)
eGFR (mL/min/1.73 m^2^)	76.5 ± 17.0
DKD (%)	32 (20.9%)
Polyneuropathy (%)	39 (25.5%)
CIMT (mm)	1.23 ± 0.55
Carotid plaque (%)	70 (45.8%)
baPWV (cm/s)	1453 ± 342
baPWV ≥ 1400 cm/s (%)	71 (46.4%)
History of CVD (%)	6 (3.9%)
10-year estimated CVD risk (%)*^a^*	14.6 ± 12.0
10-year estimated CVD risk ≥ 20%*^a^*	39 (25.5%)
Severe hypoglycemia (%)	24 (15.7%)
Hypoglycemia unawareness (%)	56 (36.6%)
DTSQ total score	27.1 ± 5.7
DTSQ item 2 (perception of hyperglycemia)	3.6 ± 1.4
DTSQ item 3 (perception of hypoglycemia)	2.6 ± 1.4

Abbreviations: baPWV, brachial–ankle pulse wave velocity; BMI, body mass index; CIMT, carotid intima–media thickness; CSII, continuous subcutaneous insulin infusion; CV, coefficient of variation; CVD, cardiovascular disease; DKD, diabetic kidney disease; DTSQ, diabetes treatment satisfaction questionnaire; eGFR, estimated glomerular filtration rate; GMI, glucose management indicator; HbA1c, glycated hemoglobin; LDL-C, low-density lipoprotein cholesterol; rtCGM, real-time continuous glucose monitoring; SGLT2, sodium–glucose cotransporter 2; TAR, time above range; TBR, time below range; TG, triglyceride; TIR, time in range; UACR, urine albumin-to-creatinine ratio.

Categorical data are expressed as n (%) and continuous data as mean ± SD or geometric mean (interquartile range).

Antihypertensive drugs include angiotensin-converting enzyme inhibitors, angiotensin receptor blockers, and calcium antagonists.

^*a*^n = 147.

Cluster analysis classified all patients into four clusters, with an average silhouette width of 0.51. Figure 1 illustrates a boxplot comparing the clusters. Cluster 1 (n = 53) exhibited good glycemic control, showing a mean GMI of 6.7 ± 0.3%, mean Glucose CV of 31.7 ± 3.0%, and mean TIR_70-180 mg/dL_ of 79.8 ± 5.5%. Cluster 2 (n = 46) demonstrated large glycemic variability with hyperglycemia, showing a mean GMI of 7.4 ± 0.3%, mean Glucose CV of 35.2 ± 3.6%, mean TIR_70-180 mg/dL_ of 58.0 ± 7.8%, and mean TAR_>180 mg/dL_ of 40.4 ± 7.7%. Cluster 3 (n = 39) demonstrated large glycemic variability with hypoglycemia, showing a mean GMI of 6.7 ± 0.2%, mean Glucose CV of 42.0 ± 4.2%, mean TIR_70-180 mg/dL_ of 69.2 ± 7.5%, and mean TBR_<70 mg/dL_ of 8.3 ± 3.5%. Cluster 4 (n = 15) demonstrated large glycemic variability with hyperglycemia and hypoglycemia, showing a mean GMI of 8.4 ± 0.9%, mean Glucose CV of 40.1 ± 8.1%, mean TIR_70-180 mg/dL_ of 38.3 ± 10.0%, mean TAR_>180 mg/dL_ of 58.3 ± 12.0%, and mean TBR_<54 mg/dL_ of 1.1 ± 1.5%. As shown in the appendix (28), cluster stability in the present study was generally robust and reproducible.

**Figure 1:**
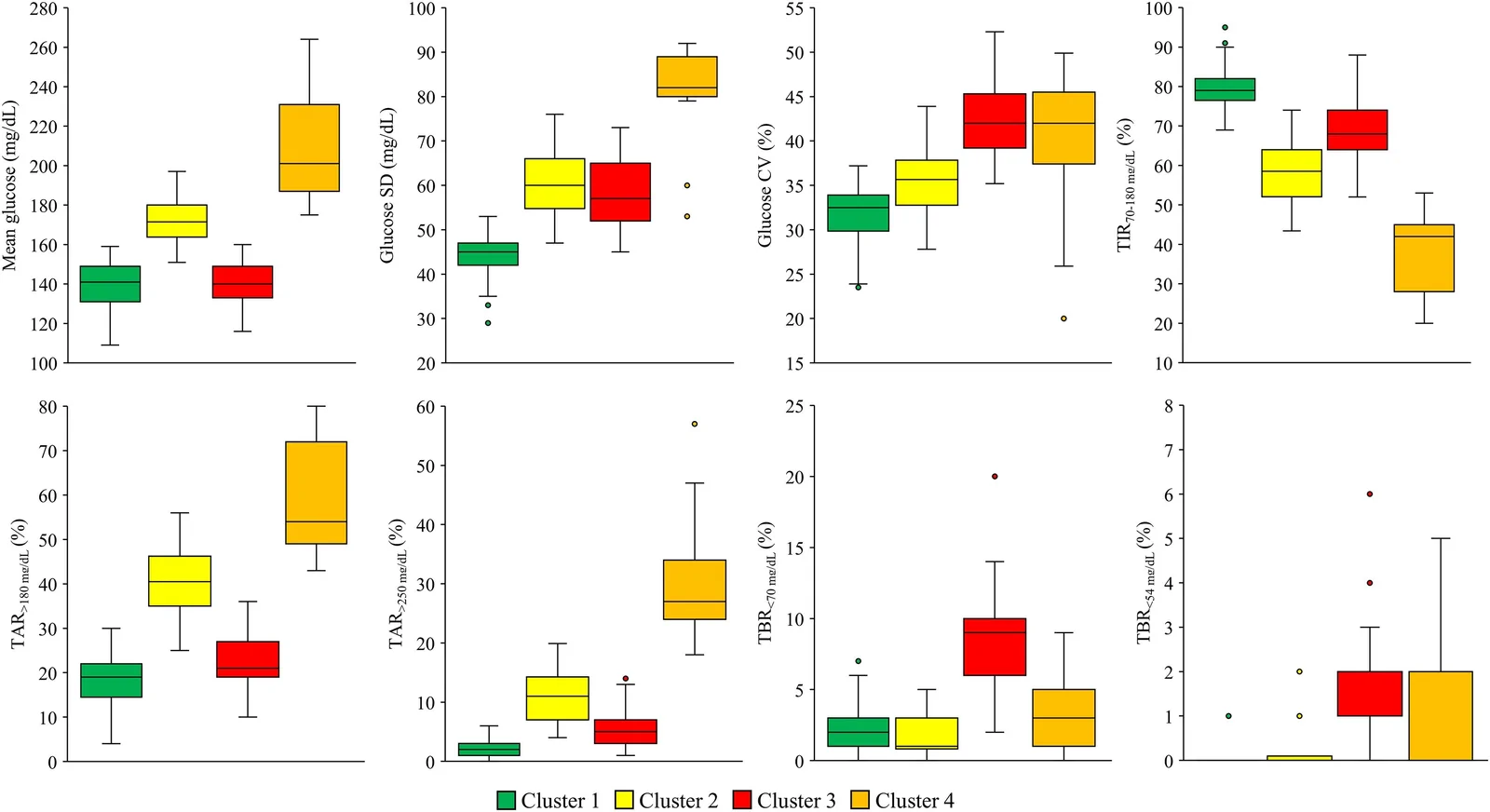
Distribution of mean glucose, glucose SD, glucose CV, TIR_70-180 mg/dL_, TAR_>180 mg/dL_, TAR_>250 mg/dL_, TBR_<70 mg/dL_, and TBR_<54 mg/dL_ for each cluster. Dots represent outliers. CV, coefficient of variation; TAR, time above range; TBR, time below range; TIR, time in range.

Table 2 compares patient characteristics and diabetic complications across clusters, with Cluster 1 as the control group. Cluster 1 exhibited a lower prevalence of current smoking, a higher likelihood of using rtCGM and CSII, and higher C-peptide levels than the other clusters. Clusters 2 and 4 demonstrated higher HbA1c levels than Cluster 1. Cluster 4 had a higher systolic blood pressure, higher likelihood of using SGLT2 inhibitors, and higher score of DTSQ item 2 than Cluster 1. Before adjusting for covariates, Cluster 2 had a higher prevalence of elevated baPWV compared with Cluster 1 (34.0% vs 56.5%, *P* < .05). Cluster 3 had a higher prevalence of polyneuropathy and severe hypoglycemia than Cluster 1 (17.0% vs 35.9% and 9.4% vs 35.9%, respectively; all *P* < .05). Cluster 4 had a higher prevalence of polyneuropathy, elevated baPWV, and elevated CVD risk scores than Cluster 1 (17.0% vs 46.7%; 34.0% vs 66.7%; and 15.1% vs 46.7%, respectively; all *P* < .05).

**Table 2:** Comparison of patient characteristics among the four clusters

	Reference			
Parameters	Cluster 1 (n = 53)	Cluster 2 (n = 46)	Cluster 3 (n = 39)	Cluster 4 (n = 15)
Age (years)	48.6 ± 12.6	46.0 ± 13.7	51.1 ± 14.1	50.1 ± 14.4
Male (%)	20 (37.7%)	20 (43.5%)	19 (48.7%)	7 (46.7%)
Duration of diabetes (years)	13.7 ± 11.2	17.4 ± 11.1	17.1 ± 14.8	14.3 ± 8.3
Type 1 diabetes classification (acute-onset/slowly progressive/fulminant)	38/10/5	28/15/3	25/8/6	11/2/2
Current smoking (%)	3 (5.7%)	11 (23.9%)*^a^*	10 (25.6%)*^a^*	4 (26.7%)*^a^*
Use of rtCGM (%)	50 (94.3%)	30 (65.2%)*^a^*	19 (48.7%)*^a^*	8 (53.3%)*^a^*
Use of CSII (%)	44 (83.0%)	28 (60.9%)*^a^*	19 (48.7%)*^a^*	1 (6.7%)*^a^*
BMI (kg/m^2^)	23.1 ± 4.0	23.6 ± 4.1	23.0 ± 3.8	25.1 ± 4.3
LDL-C (mg/dL)	107.4 ± 25.0	113.0 ± 26.1	109.0 ± 30.0	110.8 ± 29.9
TG (mg/dL)	70.0 (54.5-101.5)	70.0 (56.5-107.5)	71.0 (52.0-107.0)	84.0 (67.0-140.0)
Statin (%)	20 (37.7%)	12 (26.1%)	12 (30.8%)	3 (20.0%)
Systolic blood pressure (mmHg)	125.5 ± 18.5	126.4 ± 16.6	130.3 ± 18.0	139.0 ± 17.1*^a^*
Antihypertensives (%)	14 (26.4%)	6 (13.0%)	6 (15.4%)	2 (13.3%)
SGLT2 inhibitors (%)	2 (3.8%)	7 (15.2%)	3 (7.7%)	4 (26.7%)*^a^*
C-peptide (ng/mL)	0.18 ± 0.31	0.06 ± 0.15*^a^*	0.05 ± 0.19*^a^*	0.08 ± 0.13*^a^*
HbA_1c_ (%)	6.8 ± 0.5	7.5 ± 0.6*^a^*	6.9 ± 0.7	8.3 ± 0.9*^a^*
GMI (%)	6.7 ± 0.3	7.4 ± 0.3*^a^*	6.7 ± 0.2	8.4 ± 0.9*^a^*
Mean glucose (mg/dL)	139.4 ± 12.2	171.9 ± 10.7*^a^*	140.2 ± 10.0	206.7 ± 26.2*^a^*
Glucose SD (mg/dL)	44.3 ± 4.8	60.6 ± 7.0*^a^*	59.0 ± 7.4*^a^*	81.2 ± 11.1*^a^*
Glucose CV (%)	31.7 ± 3.0	35.2 ± 3.6*^a^*	42.0 ± 4.2*^a^*	40.1 ± 8.1*^a^*
TIR_70-180 mg/dL_ (%)	79.8 ± 5.5	58.0 ± 7.8*^a^*	69.2 ± 7.5*^a^*	38.3 ± 10.0*^a^*
TAR_>180 mg/dL_ (%)	18.0 ± 6.2	40.4 ± 7.7*^a^*	22.5 ± 6.7*^a^*	58.3 ± 12.0*^a^*
TAR_>250 mg/dL_ (%)	2.2 ± 1.3	11.0 ± 4.5*^a^*	5.6 ± 3.3*^a^*	29.8 ± 10.4*^a^*
TBR_<70 mg/dL_ (%)	2.3 ± 1.7	1.6 ± 1.4	8.3 ± 3.5*^a^*	3.4 ± 3.1
TBR_<54 mg/dL_ (%)	0.1 ± 0.3	0.2 ± 0.5	1.8 ± 1.3*^a^*	1.1 ± 1.5*^a^*
Retinopathy (%)	12 (22.6%)	17 (37.0%)	6 (15.4%)	1 (6.7%)
UACR (mg/g)	4.9 (3.6-9.1)	5.8 (3.3-12.9)	7.9 (4.2-17.9)*^a^*	6.6 (4.4-11.7)
eGFR (mL/min/1.73 m^2^)	70.6 ± 13.4	79.7 ± 19.0*^a^*	77.5 ± 17.6*^a^*	84.5 ± 14.9*^a^*
DKD (%)	12 (22.6%)	9 (19.6%)	10 (25.6%)	1 (6.7%)
Polyneuropathy (%)	9 (17.0%)	9 (19.6%)	14 (35.9%)*^a^*	7 (46.7%)*^a^*
CIMT (mm)	1.16 ± 0.54	1.17 ± 0.48	1.25 ± 0.54	1.59 ± 0.71*^a^*
Carotid plaque (%)	21 (39.6%)	19 (41.3%)	20 (51.3%)	10 (66.7%)
baPWV (cm/s)	1391 ± 288	1457 ± 378	1479 ± 346	1591 ± 379*^a^*
baPWV ≥ 1400 cm/s (%)	18 (34.0%)	26 (56.5%)*^a^*	17 (43.6%)	10 (66.7%)*^a^*
History of CVD (%)	3 (5.7%)	1 (2.2%)	2 (5.1%)	0 (0.0%)
10-year estimated CVD risk (%)*^b^*	11.8 ± 7.9	13.3 ± 9.9	18.7 ± 17.4*^a^*	17.8 ± 11.0*^a^*
10-year estimated CVD risk ≥ 20%*^b^*	8 (15.1%)	12 (26.1%)	12 (30.8%)	7 (46.7%)*^a^*
Severe hypoglycemia (%)	5 (9.4%)	4 (8.7%)	14 (35.9%)*^a^*	1 (6.7%)
Hypoglycemia unawareness (%)	19 (35.8%)	14 (30.4%)	19 (48.7%)	4 (26.7%)
DTSQ total score	27.4 ± 6.1	26.2 ± 5.5	27.9 ± 5.8	26.3 ± 4.8
DTSQ item 2 (perception of hyperglycemia)	3.4 ± 1.4	3.7 ± 1.2	3.1 ± 1.5	4.6 ± 1.5*^a^*
DTSQ item 3 (perception of hypoglycemia)	2.6 ± 1.3	2.4 ± 1.4	3.0 ± 1.4	2.3 ± 1.4

Abbreviations: baPWV, brachial–ankle pulse wave velocity; BMI, body mass index; CIMT, carotid intima–media thickness; CSII, continuous subcutaneous insulin infusion; CV, coefficient of variation; CVD, cardiovascular disease; DKD, diabetic kidney disease; DTSQ, diabetes treatment satisfaction questionnaire; eGFR, estimated glomerular filtration rate; GMI, glucose management indicator; HbA_1c_, glycated hemoglobin; LDL-C, low-density lipoprotein cholesterol; rtCGM, real-time continuous glucose monitoring; SGLT2, sodium–glucose cotransporter 2; TAR, time above range; TBR, time below range; TG, triglyceride; TIR, time in range; UACR, urine albumin-to-creatinine ratio.

Categorical data are expressed as n (%) and continuous data as mean ± SD or geometric mean (interquartile range).

Antihypertensive drugs include angiotensin-converting enzyme inhibitors, angiotensin receptor blockers, and calcium antagonists.

^*a*^ *P* < .05.

^*b*^n = 50/45/37/15.

Table 3 compares the risk of diabetic complications across clusters based on the logistic regression analysis results, with Cluster 1 as the control group. After adjustment for age, sex, and duration of diabetes, Cluster 2 had a higher risk of elevated baPWV (aOR: 5.38 [95%CI, 1.73-16.7]), and Cluster 3 had a higher risk of severe hypoglycemia (aOR: 6.11 [1.68-22.3]). Cluster 4 exhibited higher risks of polyneuropathy, elevated baPWV, and elevated CVD risk scores (aOR: 4.87 [1.20-19.7], 7.58 [1.45-39.7], and 20.2 [1.57-260.5], respectively). Table S3 (28) presents the results of analyses with additional covariate adjustment for outcomes other than the CVD risk score, and Table S4 (28) presents analyses examining the associations between CIMT or baPWV and individual glycemic variability metrics.

**Table 3:** Comparison of the risk of diabetic complications by cluster

Outcome	Cluster 1 (n = 53)		Cluster 2 (n = 46)		Cluster 3 (n = 39)		Cluster 4 (n = 15)	
	aOR*^a^* (95%CI)	*P*	aOR*^a^* (95%CI)	*P*	aOR*^a^* (95%CI)	*P*	aOR*^a^* (95%CI)	*P*
Retinopathy								
Model 1	Reference	—	2.00 (0.83-4.82)	.121	0.62 (0.21-1.83)	.388	0.24 (0.03-2.05)	.194
Model 2	Reference	—	1.68 (0.59-4.79)	.333	0.28 (0.07-1.19)	.086	0.23 (0.02-2.18)	.200
DKD								
Model 1	Reference	—	0.83 (0.31-2.20)	.709	1.18 (0.45-3.09)	.739	0.24 (0.03-2.05)	.194
Model 2	Reference	—	0.69 (0.24-2.00)	.500	0.82 (0.28-2.38)	.714	0.17 (0.02-1.54)	.115
Polyneuropathy								
Model 1	Reference	—	1.19 (0.43-3.31)	.740	2.74 (1.04-7.23)	.042	4.28 (1.24-14.8)	.022
Model 2	Reference	—	1.22 (0.39-3.78)	.737	2.39 (0.82-7.01)	.112	4.87 (1.20-19.7)	.027
Carotid plaque								
Model 1	Reference	—	1.07 (0.48-2.40)	.865	1.60 (0.70-3.70)	.267	3.05 (0.91-10.2)	.070
Model 2	Reference	—	1.20 (0.44-3.24)	.723	1.23 (0.46-3.33)	.681	4.27 (0.89-20.4)	.069
baPWV ≥ 1400 cm/s								
Model 1	Reference	—	2.53 (1.12-5.71)	.026	1.50 (0.64-3.52)	.348	3.89 (1.15-13.1)	.028
Model 2	Reference	—	5.38 (1.73-16.7)	.004	1.12 (0.38-3.34)	.838	7.58 (1.45-39.7)	.017
10-year estimated CVD risk ≥20%*^b^*								
Model 1	Reference	—	1.91 (0.70-5.21)	.207	2.52 (0.91-7.01)	.076	4.59 (1.30-16.3)	.018
Model 2	Reference	—	3.54 (0.56-22.5)	.181	2.86 (0.52-15.9)	.229	20.2 (1.57-260.5)	.021
Severe hypoglycemia								
Model 1	Reference	—	0.91 (0.23-3.63)	.899	5.38 (1.74-16.6)	.004	0.69 (0.07-6.37)	.740
Model 2	Reference	—	0.77 (0.17-3.38)	.726	6.11 (1.68-22.3)	.006	0.81 (0.08-8.23)	.859

Abbreviations: aOR, adjusted odds ratio; baPWV, brachial–ankle pulse wave velocity; CI, confidence interval; CVD, cardiovascular disease; DKD, diabetic kidney disease.

^*a*^Model 1 was not adjusted; Model 2 was adjusted for age, sex, and duration of diabetes.

^*b*^n = 33/9/35/48.

## Discussion

This study identified four distinct clusters of glycemic profiles among Japanese patients with type 1 diabetes using cluster analysis of CGM data. These clusters demonstrated varying risks of diabetic complications. Cluster 2, characterized by large glycemic variability with hyperglycemia, had a higher risk of elevated baPWV. Cluster 3, characterized by large glycemic variability with hypoglycemia, had a higher risk of severe hypoglycemia. Cluster 4, characterized by the greatest glycemic variability associated with hyperglycemia and hypoglycemia, exhibited higher risks of polyneuropathy, elevated baPWV, and elevated CVD risk scores.

Recent studies have increasingly reported associations between CGM-derived metrics and diabetic complications in patients with type 1 diabetes. With respect to microangiopathy, indicators such as TIR have been shown to be associated with retinopathy, UACR, DKD, and neuropathy (3-6). Similarly, associations have been reported between CGM metrics and surrogate markers of atherosclerosis, including CIMT and pulse wave velocity (7). Similar to the present study, a previous investigation that classified patients with type 1 diabetes into three distinct glycemic profiles based on CGM data demonstrated an increased risk of retinopathy and DKD in individuals with hyperglycemia or hypoglycemia (9). Consistent with these findings, the present study demonstrated that Cluster 2, characterized by marked hyperglycemia and large glycemic variability, was associated with an increased risk of elevated baPWV. Furthermore, Cluster 4, which exhibited the greatest glycemic variability associated with hyperglycemia and hypoglycemia, showed significantly higher risks of polyneuropathy and elevated baPWV. Although the risk of carotid plaque was also relatively high in Cluster 4, statistical significance was not achieved. The results of analyses with additional covariate adjustment (Table S3 (28)) and analyses examining the associations between CIMT or baPWV and glycemic variability metrics (Table S4 (28)) were generally consistent with the findings of the primary analysis. However, it should be noted that the associations in Cluster 4 were no longer statistically significant after additional adjustment, likely in part because of the limited sample size.

Regarding the mechanisms underlying the role of glycemic variability in the development of microvascular complications, clinical studies have demonstrated that blood glucose fluctuations promote the production of oxidative stress markers, such as 8-iso prostaglandin F2alpha and 3-nitrotyrosine, to a greater extent than sustained chronic hyperglycemia (29, 30). Consistently, basic research using human coronary artery endothelial cells and human umbilical vein endothelial cells has shown that oxidative stress-induced endothelial damage is more pronounced under intermittent hyperglycemic conditions than under continuous hyperglycemia (31, 32). These findings suggest that oxidative stress and endothelial injury induced by glycemic variability contribute to the progression of diabetic complications, and the impact of glycemic variability on the development of complications may exceed that of chronic hyperglycemia alone. Nevertheless, owing to the cross-sectional design and relatively small sample size, the present study may not have fully detected the risk of some complications.

In the present cohort, only six patients (3.9%) had a history of CVD, precluding direct evaluation of CVD risk. However, calculation of CVD risk scores using the Steno Type 1 Risk Engine revealed that Cluster 4 had a higher predicted risk of CVD. Although variability in HbA1c has previously been reported as a risk factor for CVD in patients with type 1 diabetes (33), research on the association between CGM-derived metrics and CVD remains limited. A recent cross-sectional study using rtCGM demonstrated that TIR and time in tight range were associated with the presence of cerebrovascular accidents (8). Consistently, the present study suggests that patients with greater glycemic variability may have an increased latent risk of CVD. This observation supports the potential role of glycemic variability as a CVD risk factor in type 1 diabetes and suggests that the management of conventional CVD risk factors incorporated into risk score calculations (eg, smoking status and blood pressure profiles) may be suboptimal in populations with pronounced glycemic variability, such as Cluster 4.

In Cluster 3, characterized by prolonged exposure to hypoglycemia and marked glycemic variability, the risk of severe hypoglycemia was significantly elevated. This finding corroborates a previous report showing that extended duration of hypoglycemia, as assessed by CGM, was a risk factor for severe hypoglycemia (34). This prior research also identified hypoglycemia unawareness as a risk factor for severe hypoglycemia. In the present study, although no statistically significant difference was observed compared with Cluster 1, approximately half of the patients in Cluster 3 (19 individuals; 48.7%) exhibited hypoglycemia unawareness. Moreover, patients with hypoglycemia unawareness tended to have a higher risk of severe hypoglycemia even after adjustment for age, sex, and diabetes duration (aOR, 2.47; 95%CI, 0.90-6.76; *P* = .078). These findings suggest that, in Cluster 3, hypoglycemia unawareness, in addition to prolonged hypoglycemic exposure, may contribute to the increased risk of severe hypoglycemia.

Patients in Clusters 2-4, characterized by suboptimal glycemic control, require appropriate therapeutic interventions. First, the utilization rates of rtCGM and CSII, both effective for improving glycemic control (35, 36), were low in these patients. Differences in lifestyle, insufficient disease-related knowledge, and financial constraints may hinder the adoption of these devices (37, 38), highlighting the need to tailor therapeutic strategies to each patient's individual circumstances. Second, in Japan, the use of some SGLT2 inhibitors (eg, dapagliflozin) is covered by insurance for patients with type 1 diabetes. SGLT2 inhibitors have been reported to improve HbA1c levels and increase TIR in patients with type 1 diabetes (39). Given the limited availability of oral glucose-lowering agents for patients with type 1 diabetes, SGLT2 inhibitors may represent a potentially effective therapeutic option. Third, contrary to our expectations, the DTSQ scores revealed no significant differences among clusters in terms of treatment satisfaction or perceptions of hyperglycemia and hypoglycemia, although the perception of hyperglycemia (DTSQ item 2) was higher in Cluster 4 than in the reference group (Cluster 1). These findings suggest that a substantial number of patients in clusters with marked glycemic variability (Clusters 2-4) may not fully recognize the extent of prolonged hyperglycemic or hypoglycemic exposure and may perceive their current glycemic management as adequate. Accordingly, bridging the gap between healthcare providers’ assessments of inadequate glycemic control and patients’ perceptions is necessary for better glycemic management.

The strengths of this study include the classification of Japanese patients with type 1 diabetes into clusters with distinct glycemic variability profiles based on CGM-derived metrics and clarification of their associations with diabetic complications. However, some limitations should be acknowledged. First, owing to the cross-sectional design, causal relationships between cluster classification and diabetic complications could not be established. Although we plan to prospectively follow this cohort, longitudinal data are currently available for only a limited number of patients and are insufficient for robust longitudinal analyses. Second, the relatively small sample size may have limited the accurate assessment of the risk of certain diabetic complications. In particular, because Cluster 4 had the smallest sample size, the generalizability of the estimated complication risks may be limited. One possible explanation for the small number of patients classified into Cluster 4 is that glycemic control in patients with type 1 diabetes has improved in recent years because of advances in devices such as CGM and CSII (40). Third, because only eight CGM-derived metrics were included in our study, other indices of glycemic variability—such as mean amplitude of glycemic excursions and time in tight range—could not be evaluated. In addition, differences in glycemic variability according to time periods, such as daytime vs nighttime, were not assessed. The selected metrics were chosen because they represent major domains of glycemic control, including mean glucose exposure, glycemic variability, hyperglycemia, and hypoglycemia, and are widely used in international CGM consensus recommendations. However, inclusion of additional CGM-derived metrics might have influenced the clustering results and yielded different glycemic profiles. Fourth, the study population was derived from only two medical institutions, which may have introduced selection bias. Therefore, the generalizability of the present findings may be limited.

In conclusion, Japanese patients with type 1 diabetes were classified into four distinct clusters based on CGM-derived metrics, and the risks of diabetic complications differed among these clusters. These findings support the validity of using CGM data to identify populations at high risk of diabetic complications and highlight the importance of implementing cluster-specific therapeutic strategies. Further studies, including large-scale prospective cohorts encompassing a broader spectrum of glycemic profiles, are warranted to determine whether these clusters are the optimal classification.

## Data Availability

Some or all datasets generated during and/or analyzed during the current study are not publicly available but are available from the corresponding author on reasonable request.
